# Neoadjuvant PD-1 Blockade Combined With Chemotherapy Followed by Concurrent Immunoradiotherapy in Locally Advanced Anal Canal Squamous Cell Carcinoma Patients: Antitumor Efficacy, Safety and Biomarker Analysis

**DOI:** 10.3389/fimmu.2021.798451

**Published:** 2022-01-14

**Authors:** WeiWei Xiao, Yan Yuan, SuiHai Wang, Zhidong Liao, PeiQiang Cai, BaoQing Chen, Rong Zhang, Fang Wang, ZhiFan Zeng, YuanHong Gao

**Affiliations:** ^1^ Department of Radiation Oncology, State Key Laboratory of Oncology in South China, Collaborative Innovation Center for Cancer Medicine, Sun Yat-Sen University Cancer Center, Guangzhou, China; ^2^ Institute of Antibody Engineering, School of Laboratory Medicine and Biotechnology, Southern Medical University, Guangzhou, China; ^3^ Department of Pathology, Meizhou Hospital of Traditional Chinese Medicine, Meizhou, China; ^4^ Departments of Medical Imaging and Interventional Radiology, State Key Laboratory of Oncology in South China, Collaborative Innovation Center for Cancer Medicine, Sun Yat-Sen University Cancer Center, Guangzhou, China; ^5^ Department of Endoscopy, State Key Laboratory of Oncology in South China, Collaborative Innovation Center for Cancer Medicine, Sun Yat-Sen University Cancer Center, Guangzhou, China; ^6^ Department of Molecular Diagnosis, State Key Laboratory of Oncology in South China, Collaborative Innovation Center for Cancer Medicine, Sun Yat-Sen University Cancer Center, Guangzhou, China

**Keywords:** locally advanced, anal canal squamous cell carcinoma, neoadjuvant, PD-1 blockade, PD-L1

## Abstract

**Background:**

Anal canal squamous cell carcinoma (ACSCC) is an exceedingly rare malignant neoplasm with challenges in sphincter preservation, treatment toxicities and long-term survival. Little is known concerning the activity of PD-1 antibodies in locally advanced ACSCC. This study reports on the efficacy and toxicities of a neoadjuvant PD-1 blockade combined with chemotherapy followed by concurrent immunoradiotherapy in ACSCC patients, and describes biomarkers expression and mutation signatures.

**Methods:**

In this cohort study, patients were treated as planned, including four cycles of neoadjuvant PD-1 antibody toripalimab combined with docetaxol and cisplatin, followed by radiotherapy and two cycles of concurrent toripalimab. Multiplex immunofluorescence staining (mIHC) with PD-L1, CD8, CD163, Pan-Keratin and DAPI was performed with the pretreatment tumor tissue. Whole exome sequencing was performed for the primary tumor and peripheral blood mononuclear cells. The primary endpoint was the complete clinical response (cCR) rate at 3 months after overall treatment. Acute and late toxicities graded were assessed prospectively.

**Results:**

Five female patients with a median age of 50 years old (range, 43-65 years old), finished treatment as planned. One patient had grade 3 immune related dermatitis. Two patients had grade 3 myelosuppression during neoadjuvant treatment. No severe radiation-related toxicities were noted. Four patients with PD-L1 expression >1% achieved a cCR after neoadjuvant treatment. and the other patient with negative PD-L1 expression also achieved a cCR at 3 months after radiotherapy. All the patients were alive and free from disease and had a normal quality of life, with 19.6-24 months follow up. Inconsistent expression of PD-L1 and CD163 was detected in 3 and 5 patients, respectively. TTN, POLE, MGAM2 were the top mutation frequencies, and 80 significant driver genes were identified. Pathway analysis showed enrichment of apoptosis, Rap1, Ras, and pathways in cancer signaling pathways. Eight significantly deleted regions were identified.

**Conclusions:**

This small cohort of locally advanced ACSCC patients had quite satisfactory cCR and sphincter preservation rate, after neoadjuvant PD-1 antibody toripalimab combined with chemotherapy followed by concurrent immunoradiotherapy, with mild acute and long-term toxicities.

## Introduction

In the worldwide, anal canal squamous cell carcinoma (ACSCC) is a rare malignant tumor with increasing incidence (1). The United Kingdom Coordinating Committee on Cancer Research (UKCCCR) randomized ACT I trial and the European Organization for Research and Treatment of Cancer (ETROC) phase III randomized trial established concurrent chemoradiotherapy (CRT) as the standard treatment for locoregional ACSCC (2, 3). The complete clinical response (cCR) rate was noted to be 52%, 71%, and 78% at 11, 18, and 26 weeks from the start of CRT, respectively (4). The rate of severe acute hematologic toxicity (grade 3 and 4) was 42%-61%, and the rate of severe late toxic effects was 10%-11% in RTOG 9811 trial (5). Although chemotherapy combined with radiotherapy has reduced the absolute risk of locoregional relapse and decreased the colostomy rate, there are still about 10-30% of patients would have disease progression and 25-40% patients would have colostomy in 3 years from the initial diagnosis (4, 6). Therefore, adding other agents and modifying treatment strategy might be meaningful for better disease control, lower need for colostomy and less treatment toxicities.

Meanwhile, PD-1 monoclonal antibody has shown a good anti-tumor response in various solid carcinomas, including metastatic ACSCC (7, 8). PD-1 inhibitors including pembrolizumab and nivolumab have been approved for the treatment of metastatic anal squamous cell carcinoma (9). Nevertheless, the role of PD-1 monoclonal antibody in locoregional disease of anal squamous cell carcinoma is still under active investigation by the American National Cancer Institute with no results have been reported (NCT03233711, NCT04719988). We speculated that PD-1 monoclonal antibody may have important value in disease control and organ preservation by increasing the rate of cCR in ACSCC. In the present study, we explored the efficacy and safety of the PD-1 monoclonal antibody toripalimab for the locoregional ACSCC in the neoadjuvant setting and the mode of combination with radiotherapy.

## Methods

### Population

From July 2019 to December 2019, all newly diagnosed locally advanced ACSCC patients at Sun Yat-sen University Cancer Center were treated as plan as a pilot study. Totally, five locally advanced ACSCC patients (T2-4, N0 or Any T, N+) received chemoradioimmunotherapy. The details of the enrolled patients are shown in **Table 1**. Their median age was 50 (range, 43-65) years at diagnosis. All the five patients were female. Three patients had stage II disease while two patients had stage III disease. Maximum diameters of the primary tumors range from 20-55mm.

**Table 1:** Clinical information and biomarker of the 5 locally advanced ACSCC patients.

Patient Number		1	2	3	4	5
Age		65	50	51	44	43
Gender		female	female	female	female	female
Tumor maximum diameter (mm)		37	33	20	40	55
Clinical TNM stage		T2N0M0	T4N0M0	T2N0M0	T3N0M0	T3N1M0
Involved Structures		sphincter	vagina	none	none	sphincter
TMB		1	4	38	7	50
TNB		1	6	3	7	58
Tumor	PD-L1+	93.8%	1.2%	9.8%	2.4%	0.3%
	CD8+	0	0	28.5%	21.0%	9.1%
	CD163+	7.8%	6.1%	22.3%	16.9%	10.5%
Paracancerous stroma	PD-L1+	0	0.3%	0.1%	0.2%	0
	CD8+	0	0	30.4%	38.0%	12.3%
	CD163+	71.9%	30.1%	19.7%	6.7%	7.7%
Response after neoadjuvant treatment		cCR	cCR	cCR	cCR	near CR
Response after radiotherapy		cCR	cCR	cCR	cCR	cCR
Grade 3 irAE		none	dermatitis	none	none	none

ACSCC, anal canal squamous cell carcinoma; TNM, tumor node metastasis; TMB, tumor mutational burden; TNB, tumor neoantigen burden; irAE, immune related adverse event; DFS, disease free survival.

### Study Design

Docetaxol (75mg/m^2^, d1), cisplatin (37.5 mg/m^2^, d2-3), and PD-1 antibody toripalimab (240mg, d1) were given every three weeks. Four cycles of neoadjuvant treatment were planned and four patients completed all the cycles. One patient completed only three cycles of chemotherapy and toripalimab and one cycle of chemotherapy alone due to grade 3 dermatitis caused by the PD-1 blockade. Next, all five patients received definitive radiotherapy. Three patients received two cycles of concurrent toripalimab every three weeks and the other two patients received radiotherapy without concurrent medication due to treatment gain concern. According to the Radiation Therapy Oncology Group (RTOG) 0529, the target volumes were contoured on the planning CT slices (10). The gross tumor volume (GTV) was defined as the primary tumor and metastatic lymph nodes according to the diagnosis image with 50 Gy/25 fractions. The clinical target volume 1 (CTV1) was defined as the GTV with an expansion of 2.5cm to the primary tumor at diagnosis image, and 1cm to metastatic lymph nodes at diagnosis image, and elective nodal areas (perirectal, internal and external iliac lymph node regions, presacral lymph node regions). The dose of CTV1 was 45Gy/25 fractions. The bilateral inguinal lymph node regions were defined as the clinical target volume 2 (CTV2). The dose of CTV2 was 42.5Gy/25 fractions. The planning target volumes (PTVs) were created based on GTV and CTVs with a 0.6cm expansion. All patients were evaluated using MRI imaging, colonoscopy and digital examination after every two cycles of neoadjuvant treatment, and regularly after definitive immunoradiotherapy according to the National Comprehensive Cancer Network (NCCN) guidelines.

Patients were assessed for tumor response using the Response Evaluation Criteria in Solid Tumors (RECIST v1.1). cCR was defined as the absence of a primary and nodal tumor by clinical examination, including digital examination, colonoscopy, and MRI of the pelvis according to the guidelines. Biopsy was needed when suspicious residual tumor exist at appropriate time point. cCR was regularly assessed during and after treatment.

Treatment-associated adverse events (AEs) were monitored throughout the study and for 30 days after treatment (90 days for serious AEs) and evaluated according to the National Cancer Institute Common Terminology Criteria for Adverse Events, version 5.0. Immune related AE (irAE) was defined according to the NCCN clinical practice guidelines for oncology management of immunotherapy-related toxicities.

### Multiplex Immunofluorescence Staining (mIHC)

An archived formalin-fixed, paraffin-embedded tumor sample or a newly obtained biopsy specimen was assessed at MEDx (Suzhou) Translational Medicine Co. Ltd central laboratory for PD-L1, CD8, Pan-Keratin, and CD163 expression. A mIHC assay was performed using the VENTANA PD-L1 (SP263), CD8 (SP57), Pan-Keratin (AE1/AE3/CK26), and Abcam CD163 (EPR19518) antibodies. Slides were also stained with DAPI for nuclear staining. Tumor and precancerous stroma were analyzed in the hematoxylin and eosin (H&E) images.

H&E images were categorized into two compartments including tumor and precancerous stroma by an experienced histopathologist for positivity calculation, respectively. Multi-spectral images were scanned using the quantitative pathological imaging system PerkinElmer Vectra 3.0, and analyzed using the inform software for staining positivity. Thresholds for antibody positivity were calibrated for each individual slide, and automated cell counting was utilized. Staining positivity was analyzed for each slide and for each marker. PD-L1 positivity was defined as membrane staining of ≥1% of scorable cells, including both neoplastic cells and contiguous mononuclear inflammatory cells.

### Whole Exome Sequencing (WES)

DNA was extracted from five ACSCC tumors and paired peripheral blood samples for a total of 10 samples that were used for library preparation using the Agilent SureSelect Human All Exon v7 exome capture kit and sequenced across three flow cells on the Illumina HiSeq 4000 Platform (Illumina, San Diego, CA, USA). Reads were trimmed for adapters and low-quality bases using Trimmomatic software before alignment to the human hg19 reference genome using Burrows-Wheeler Aligner (BWA) mapping software (v. 0.7.15). Mapped reads were then de-duplicated using Picard tools (v. 1.119), followed by re-alignment, and base quality score recalibration using the Genome Analysis Toolkit (GATK) (v. 4.1.3.0).

### Somatic Variant Analysis

Variant calling was performed using Strelka (v2.9.2) in tumor-normal mode following the best practice guidelines for exome-seq analysis provided by the Genome Atlas Toolkit authors (11, 12). Variants were filtered using Ensembl’s Variant Effect Predictor (VEP v. 92) and converted into a Mutation Annotation Format (MAF) using the vcf2maf tool (v. 1.6.16). Visualization and summarization were performed using custom scripts in R (v. 3.6.3), primarily utilizing the maftools packages (v. 1.8.10) for data summarization and GenVisR (v. 1.40.0) for generating plots (13). We carried out significance analysis of driver genes using the MutSigCV (1.41) algorithm. For MutSigCV (14), p-values could not be adjusted due to the small sample size, so a raw p < 0.05 was applied. Overlapped genes that were mutated in 60% of the ACSCC samples were considered significantly mutated genes (SMGs). Tumor mutational burden (TMB) was calculated as the total count of nonsynonymous mutations in the coding sequence using maftools. Tumor neoantigen burden (TNB) was calculated using NetMHCpan-4.0 (15).

### Copy Number Variant Analysis

Total copy number calls were derived using the CNVkit, and log2 scores >0.25 were considered gains, while log2 scores <-0.25 were considered losses. A significant CNV region was defined as having an amplification or deletion with a False Discovery Rate q value <0.25. Focal recurrent copy number alterations were identified using GISTIC 2.0 at a 95% confidence level (16). Visualization was performed using the svplucnv package (v. 0.9.1) in R (v. 3.6.3).

### Mutational Signature Analysis

We used non-negative matrix factorization (NMF) to perform *de-novo* mutation signature discovery in the patient samples (17). We compared the three identified mutation signatures with the 30 in the Catalogue of Somatic Mutations in Cancer (COSMIC)(https://cancer.sanger.ac.uk/cosmic/signatures_v2.tt) using the cosine similarity metric.

### Pathway Analysis

KOBAS (v. 3.0) was used to generate annotations for driver genes based on multiple databases about pathways, diseases, and Gene Ontology (18). No mutant gene pathway was removed. The R package ggplot2 (V3.3.0) was used to visualize the pathway with a p-value less than 0.1.

### Statistical Analysis

Continuous data were expressed as median with the range. Classified variables were shown as counts and percent. The software program SPSS version 19 (SPSS Inc., Chicago, IL, USA) was used for statistical analyses.

## Results

### Treatment Response and Outcomes

**Figure 1:**
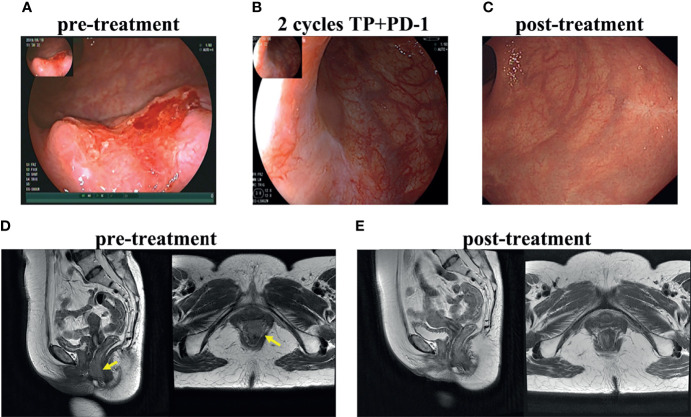
Treatment response of patient 1. Colonoscopy of primary tumor before treatment **(A)**, after 2 cycles of neoadjuvant toripalimab combined with docetaxol and cisplatin **(B)**, after radiotherapy and concurrent toripalimab **(C)**. T2WI MRI image before treatment **(D)** and after the whole treatment **(E)**.

All patients achieved cCR at 3 months after overall treatment. Even more, a cCR was achieved in patients 1, 2, 3 and 4 after neoadjuvant treatment and just before radiotherapy, while patient 5 achieved a near cCR (**Supplementary Figure 1**). Physical examination and colonoscopy showed especially rapid tumor shrinkage after the first cycle of neoadjuvant treatment in patient 1 and cCR was achieved after only two cycles of neoadjuvant treatment (**Figure 1**). All patients were alive and free from disease and had a normal quality of life, with a median 21.8 (range: 19.6-24) months follow up.

### Toxicities

Grade 3 dermatitis was noted in 1 patient which was defined as an irAE. It was relieved after corticosteroids and gamma globulin treatment. Grade 3 acute hematological toxicity occurred in two patients during neoadjuvant treatment phase. No severe radiation-related toxicities happened during radiotherapy. No patient developed severe late toxicities.

### Multiplex Immunofluorescence Staining (mIHC)

**Figure 2:**
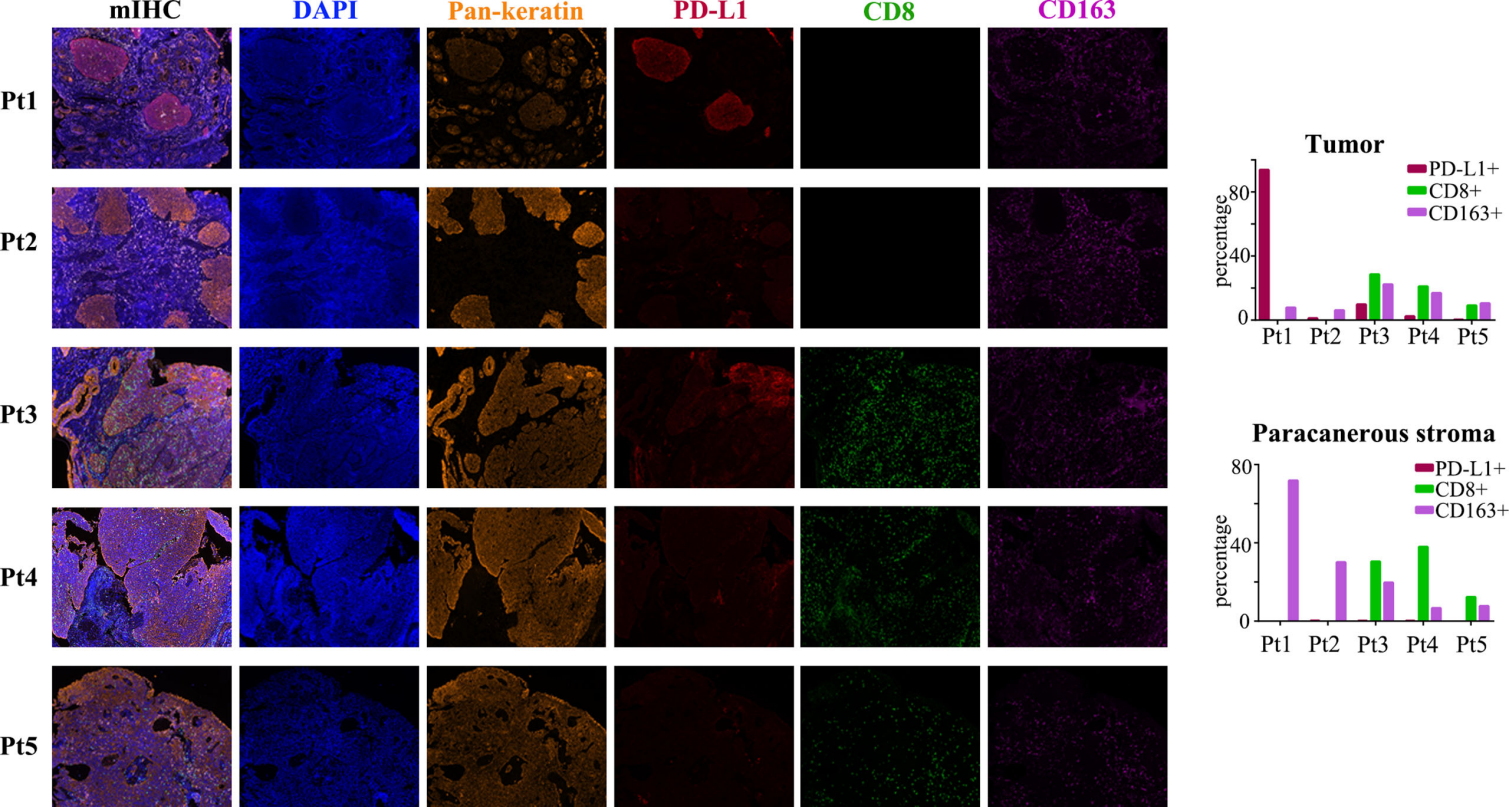
mIHC images for the five patients. Biomarkers including Pan-keratin (orange), PD-L1 (red), CD8 (green) and CD163 (purple). Expression of each PD-L1, CD8 and CD163 in the tumor and paracanerous stroma are shown in the bar charts.

To explore the tumor microenvironment (TME) of ACSCC, multiplex immunofluorescence staining was performed in pre-treatment specimens for all patients (**Figure 2**). PD-L1 positivity (≥1%) in the tumor was noted in four patients, with 1.2-93.8% expression in the tumor cells. CD8 and CD163 positivity was noted in 0.00-38.0% and 6.7-71.9% in the paracancerous stroma cells in all patients. PD-L1 expression (≥1%) in the tumor and CD163 expression in paracancerous stroma appeared positively correlated with tumor shrinkage during the neoadjuvant treatment phase. PD-L1 expression was especially high in the tumor of patient 1, with 93.8% positivity. CD163 positive cells surrounded the tumor in this patient as shown in **Figure 2**. As mentioned, patient 1 had an especially rapid tumor response which may be related to extremely high PD-L1 expression in the tumor and CD163 expression in the paracancerous stroma.

### Genomic Landscape of ACSCC

**Figure 3:**
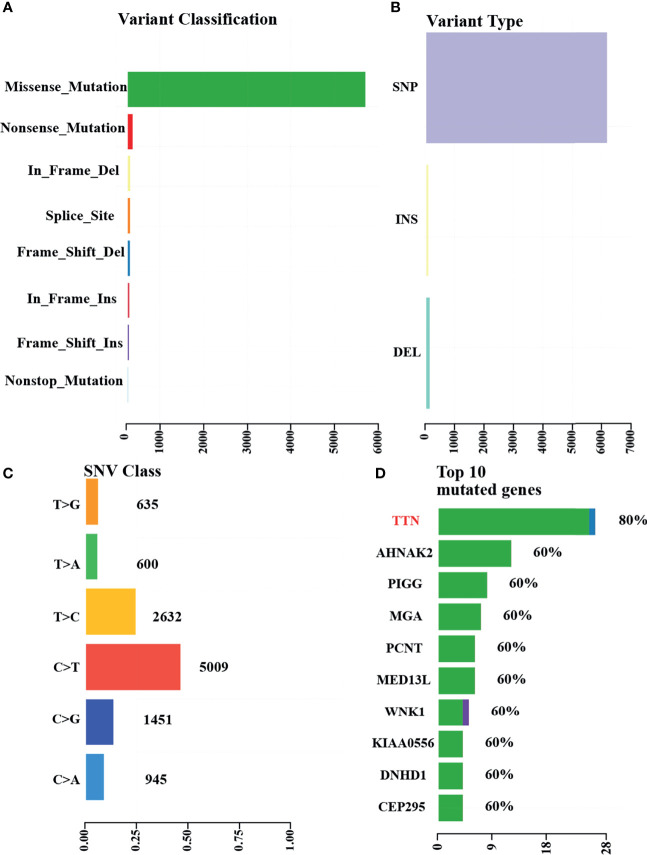
WES identified variants and top mutated genes in the five ACSCC patients. **(A)** Variant classifications. **(B)** Variants types. **(C)** SNV class. **(D)** Top 10 mutated genes. The green, blue and purple color was defined as missense_mutation,frame_shift_del and frame_shift_ins, respectively, in **Figure 3D**.

**Figure 4:**
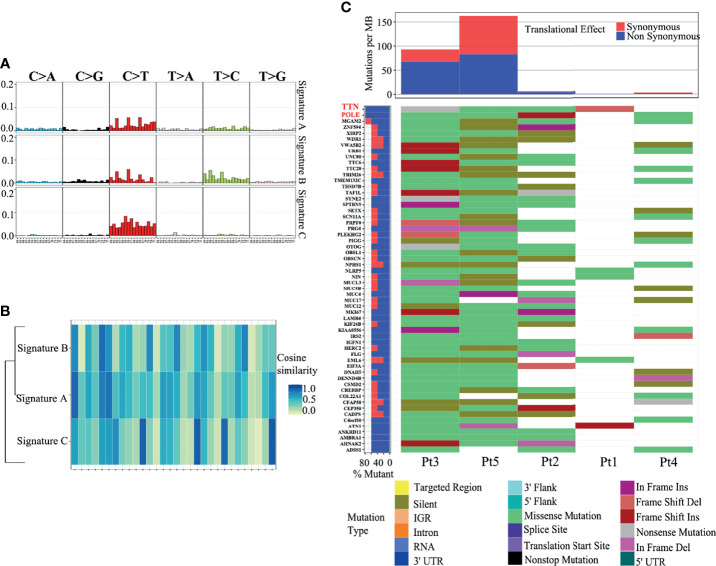
Three mutation signatures in ACSCC matched with the COSMIC database. **(A)** Nucleotide change in the three mutation signatures. **(B)** Cosine similarity of the three mutation signatures. **(C)** SMGs with a somatic mutation frequency ≥ 60% and mutation types.

To further clarify the genetic basis and seek the diagnostic markers and therapeutic targets of ACSCC, we conducted WES analysis for all the tumor specimens. The base coverage depth >30× for WES was 98% (**Supplementary Table 1**). WES identified 6,158 SNVs, with 175 indels in the exonic regions of five ACSCC samples (**Figure 3A** and **Supplementary Table 2**). The missense mutation was the main somatic single nucleotide variant (**Figure 3B**). The mutational spectrum of ACSCC was dominated by C>T/G>A transitions (**Figure 3C**). Using the BayesNMF algorithm we performed mutation signature analysis to try and disclose the ACSCC mutation processes. Three ACSCC mutation signatures were similar to three Sanger signatures in the COSMIC database (**Figures 4A, B** and **Supplementary Table 3**).

**Figure 5:**
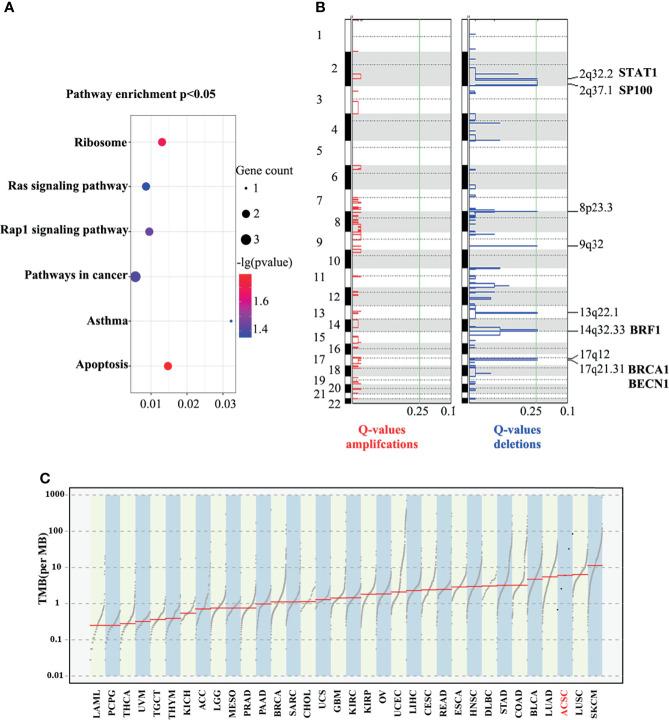
Enrichment in pathway analysis **(A)**, eight significantly deleted regions and possible lossed/deleted genes tumor suppressors **(B)**, and TMB of ACSCC and other malignant tumors **(C)**. TCGA cancer. LAML, acute myeloid leukemia; PCPG, pheochromocytoma and paraganglioma; THCA, thyroid carcinoma; UVM, uveal melanoma; TGCT, testicular germ cell tumors; KICH, kidney chromophobe; ACC, adrenocortical carcinoma; LGG, brain lower grade glioma; MESO, mesothelioma; PRAD, prostate adenocarcinoma; BRCA, breast invasive carcinoma; SARC, sarcoma; CHOL, cholangiocarcinoma; UCS, uterine carcinosarcoma; GBM, glioblastoma multiforme; KIRC, kidney renal clear cell carcinoma; OV, ovarian serous cystadenocarcinoma; UCEC, uterine corpus endometrial carcinoma; LIHC, liver hepatocellular carcinoma; CESC, cervical squamous cell carcinoma and endocervical adenocarcinoma; READ, rectum adenocarcinoma; ESCA, esophageal carcinoma; HNSC, head and neck squamous cell carcinoma; DLBC, lymphoid neoplasm diffuse large b-cell lymphoma; STAD, stomach adenocarcinoma; COAD, colon adenocarcinoma; BLCA, bladder urothelial carcinoma; LUAD, lung adenocarcinoma; LUSC, lung squamous cell carcinoma; SKCM, skin cutaneous melanoma.

We identified 55 SMGs with a somatic mutation frequency ≥ 60% in all cases. TTN, POLE, MGAM2 were the top ACSC mutation frequencies (**Figures 3D**, **4C**). Using the MutSigCV 1.41, we identified 80 significant driver genes (SDGs) (p<0.5; **Supplementary Table 4**). Pathway analysis showed enrichment of apoptosis (P = 0.016), Rap1 (P = 0.035), Ras (P = 0.042), and pathways in cancer (p=0.039) signaling pathways (**Supplementary Table 5**, **Figure 5A**).

The GISTIC algorithm was applied to identify recurrent focal somatic copy number alterations (SCNAs). Eight significantly deleted regions included 2q32.2, 2q37.1, 8p23.3, 9q32, 13q22.1, 14q32.33, 17q21.31 and 17q12 (q<0.25; **Figure 5B**), which encode well-known tumor suppressors such as STAT1, SP100, BRF1, BRCA1 and BECN1, as screened using the tumor suppressor gene database (TSGene) (**Supplementary Table 6**) (19, 20). Coincidentally, all these genes have been reported to provide immunity against virus infection, especially HPV, which is a well-known pathogenic factor in ACSCC (21). The literature also reports that they have key immune effector functions in cancer progression and response to anti-cancer treatment (22). However, no significant amplified regions were identified.

Based on the WES data, we observed a large discrepancy in TNB and TMB between five samples, ranging from one to over 50 (**Supplementary Figure 2B**). We further compared the TMB between ACSCC and pan-cancer data of The Cancer Genome Atlas (TCGA) dataset using the tcgaCompare function in the maftools R package. **Figure 5C** revealed that TMB of ACSCC ranks at the forefront among cancers, which may be the reason for the good response of ACSCC to immunotherapy, but tumor shrinkage in the neoadjuvant phase appeared unrelated with TMB, nor TNB in our cohort.

## Discussion

Concurrent chemoradiotherapy (CCRT) remains the standard treatment for non-metastatic ACSCC patients, but there are still unsolved issues. Local resistance and recurrence are the main failure pattern, which is about 40%, this then results in eventual loss of sphincter function in about 25-40% of ACSCC patients (6, 23, 24). Grade 3 and above late toxicities occurred up to 33.3%, including faecal incontinence, diarrhea and ulceration, etc (25). A large sized tumor is also correlated with a higher incidence of acute and late toxicities, causing a significantly negative impact on patients’ quality of life (25, 26).

Vendrely et al. reported in 2021 European Society for therapeutic Radiation and Oncology meeting that, adding Panitumumab to chemoradiotherapy didn’t result in expected cCR rate for locally advanced anal cancer, and furthermore a significant toxicity was documented when Panitumumab was used with chemoradiotherapy together (27). Thus, how to increase downstaging and especially cCR rate is still of vital importance for locally advanced ACSCC patients. Ideal radiation dose is also an undetermined issue in the ACSCC for satisfactory locoregional control. There are studies trying to figure out whether different doses would suit various sized tumor in anal cancer (28). In the 2019 ASCO annual conference, Professor Robert Glynne-Jones pointed out the inconsistency of the recommended dose of radiotherapy in different countries worldwide. In Netherlands, Germany, Italy and UK, the radiotherapy doses of primary tumor were 59-64.8Gy (1.8Gy/fraction), 45-63.2Gy (1.8-2.0Gy/fraction), 50.6-55Gy (2.2Gy/fraction) and 50.4-53.2Gy (1.8Gy/fraction), respectively. In Russia, the dose was 52-58Gy (2.2Gy/fraction). Whereas, in Canada and USA, the dose was 54Gy/30fractions and 50.4-54Gy/28-30fractions, respectively. Neoadjuvant treatment has not been established in these patients due to the absence of survival benefits in the ACCORD03 and RTOG9811 studies (29), yet, some data suggests neoadjuvant treatment is useful for prolonging survival for the T4 subset of patients and downstaging for decreasing radiotherapy toxicities (30, 31).

Numerous studies have established the safety and efficacy of immunotherapy in neoadjuvant therapy for several locally advanced solid tumors, such as squamous cell carcinomas of the head and neck and esophageal squamous cell carcinomas (32, 33). Meanwhile, the immunogenicity of HPV infection in ACSCC provides a strong rationale for the combined treatment (34). However, the role of immunotherapy in the definitive setting for ACSCC is under active investigation in prospective trials, including NCT03233711 and NCT04719988. Though the results have not yet been reported. Our study shows a quite satisfactory short-term effect in the neoadjuvant setting. Rapid shrinkage of tumors can decrease the radiation dose needed and may ultimately decrease late toxicities. A high CR rate also shows promise in increasing the sphincter preservation rate, improving patients’ quality of life, and may have a positive impact on overall survival. Our team in conducting a prospective clinical trial to confirm our findings (NCT05060471).

An ideal biomarker could accurately predict the efficacy of treatment. For patients with good response to neoadjuvant treatment using chemotherapy and PD-1 inhibitor, we could consider decreasing the radiation dose or abolishing the radiotherapy to decrease radiation-related toxicity and preserve higher life quality. For patients with no response to neoadjuvant treatment, the strategy can be adjusted to avoid neoadjuvant treatment-related adverse events. The prevalence of tumor PD-L1 expression in patients with squamous cell carcinoma of the anus has been reported to be between 46% and 56% and has been associated with a significantly worse PFS, with trends towards a worse OS (35, 36). While recent research indicated that PD-L1 positive were significantly associated with higher CR rate and better DFS and OS in non-metastatic ACSCC (37). In our study, PD-L1 expression was evident in 80% of the tumor samples and showed obvious heterogeneity. CD163 was positive in the paracancerous stroma of all the tumor samples, also with obvious heterogeneity.

Along with the NCI9673 study carried out in late stage ACSCC patients (8), PD-L1 expression might be positively correlated with tumor response to the PD-1 blockade as in our study. We also found that CD163 positivity seems to have a significant impact on the effect of the PD-1 blockade in the neoadjuvant setting. The above results suggest that the occurrence of ACSCC might be related to immunosuppressive tumor microenvironment and ACSCC patients with immune-enriched infiltrates might benefit from immunotherapy. Different from the findings in the NCI9673 study (8), CD8 positive cells in the paracancerous stroma seems not affect treatment response, which implies preexisting CD8 positive immune cells may not be necessary for tumor response to the PD-1 blockade in locally advanced ACSCC.

TMB in conjunction with PD-L1 expression could be a useful biomarker for immune checkpoint blockade selection across some cancer types, such as non-small cell lung cancer (38). In analyzing the TMB of ACSCC, we found that a higher TMB compared with other tumors may be the basis for ACSCC patients to benefit from immunotherapy. However, small samples in our study could not further verify the utility of TMB as a biomarker for individualized immunotherapy guidance in ACSCC.

Based on the WES analysis, we have provided a comprehensive genomic profile of ACSCC. Signature A closely resembled COSMIC signature 1 (cosine similarity: 0.83) which is the result of an endogenous mutational process initiated by spontaneous deamination of 5-methylcytosine. This signature has been found in all cancer types and in most cancer samples and correlates with age at cancer diagnosis (39). Signature B matched COSMIC signature 5 (cosine similarity: 0.81), the etiology of which is unknown. This signature exhibits a transcriptional strand bias for T>C substitutions in the ApTpN context (39). Signature C was analogous to COSMIC signature 30. It has been observed in a small subset of breast cancers (39). Several findings have been discovered. One, different from the previously known TP53, PIK3CA, FBXW7 mutations of ACSCC, we identified novel ACSCC mutations include TTN, POLE, MGAM2 (34, 40–42). TTN mutation was identified as a predictor of improved objective response rate to immune checkpoint blockade immunotherapy in seven public clinical cohorts, including advanced cases of cutaneous squamous cell carcinoma, stomach adenocarcinoma, skin cutaneous melanoma, and lung cancer (43). Cancers harboring POLE mutations were also associated with elevated expression of several immune checkpoint genes and a favorable response to immunotherapy (44, 45). FOXL2 is a known cancer driver gene in SDGs according to the IntOGen-mutations pipeline (http://www.intogen.org/mutations/) (**Supplementary Figure 2A**) (14). Nevertheless, the clinical importance of those variants needs to be validated by the multi-omics data analysis and larger sample size study. Two, we identified high C>T/G>A mutations which are similar to other squamous cell carcinomas (46). It is conceivable that a similar mechanism may contribute to this mutation. Three, we identified eight focal regions with recurrent CNV deletions which encoded STAT1, SP100, BRF1, BRCA1 and BECN1, which are tumor suppressor genes and their loss/deletion in ACSCC may contribute to tumorigenesis, due to a defective defense to HPV and deregulation of immune functions. Pathway analysis also revealed a series of significant pathways related to tumorigenesis and tumor progression. These may be the most critical pathways involved in the development and progression of ACSCC.

Limitations of this study include its small sample size and absence of tumor expression data for analyzing together with mIHC and WES results. Moreover, the biomarker analysis was only based on the literature and few sample data with no statistical support. It is necessary to extend the follow-up time and conduct a randomized control trial to confirm the efficacy and safety of this treatment strategy.

## Conclusions

Our findings imply that the novel treatment strategy including neoadjuvant PD-1 antibody toripalimab combined with chemotherapy followed by concurrent immunoradiotherapy was favorable and safe for locoregional ACSCC, resulting in quite satisfactory cCR and sphincter preservation rate. Inconsistent expression of PD-L1 and CD163 protein were detected in tumor and paracancerous stroma, respectively. Genomic alterations in ACSCC provide insights to better understand its pathogenesis. Further data from our prospective clinical trial is expected to validate the treatment effects and enable further biomarker investigation.

## Data Availability Statement

Clinical data are available upon reasonable request. The datasets used and analyzed during this study are available from the corresponding author upon reasonable request. All the original sequencing data are uploaded into the Sequence Read Archive (accession number: SRP318823) and BioProject (accession number: PRJNA725074).

## Ethics Statement

All the patients were approved to use the PD-1 blockade as a neoadjuvant therapy before the treatment commenced. This retrospective study received full approval from the ethics committee of Sun Yat-sen University Cancer Center, China. Reference number is B2020-126.

## Author Contributions

WX and YG had full access to all the data in this study and take responsibility for the integrity of the data and the accuracy of the data analysis. WX and YG designed the study. WX and YY performed the data analysis. WX, YY, PC, BC, RZ, FW, and SW performed the data acquisition. WX and YY discussed the results and wrote the paper. WX, YY, PC, BC, RZ, ZZ, ZL, and YG provided patient samples and clinical data. All co-authors reviewed the paper.

## Funding

This work was supported by the National Natural Science Foundation of China [No. 82073329 to YG], and Chinese Society of Clinical Oncology Grand [No. Y-XD202001-0144 to WX].

## Conflict of Interest

The authors declare that the research was conducted in the absence of any commercial or financial relationships that could be construed as a potential conflict of interest.

The handling editor declared a shared affiliation, though no other collaboration, with one of the authors SW.

## Publisher’s Note

All claims expressed in this article are solely those of the authors and do not necessarily represent those of their affiliated organizations, or those of the publisher, the editors and the reviewers. Any product that may be evaluated in this article, or claim that may be made by its manufacturer, is not guaranteed or endorsed by the publisher.

## Supplementary Material

Supplementary Figure 1 Treatment responses after neoadjuvant treatment for patients 2, 3, 4 and 5. Colonoscopy and T2WI MRI images of the primary tumor before and after neoadjuvant treatment.

Supplementary Figure 2 SDGs, TMB, TNB of the five ACSCC patients. **(A)** SDGs identified using the IntOGen-mutations pipeline and MutSigCV and the common mutation gene FOXL2. **(B)** TMB, TNB of the 5 ASCS.

## Glossary

**Table:**

LAML	acute myeloid leukemia
ACC	adrenocortical carcinoma
AEs	adverse events
ACSCC	anal canal squamous cell carcinoma
BLCA	bladder urothelial carcinoma
LGG	brain lower grade glioma
BRCA	breast invasive carcinoma
BWA	Burrows-Wheeler Aligner
COSMIC	catalogue of somatic mutations in cancer
CESC	cervical squamous cell carcinoma and endocervical adenocarcinoma
CHOL	cholangiocarcinoma
COAD	colon adenocarcinoma
cCR	complete clinical response
CCRT	concurrent chemoradiotherapy
CNV	copy number variant
ESCA	esophageal carcinoma
GATK	genome analysis toolkit
GBM	glioblastoma multiforme
HNSC	head and neck squamous cell carcinoma
H&E	hematoxylin and eosin
KICH	kidney Chromophobe
KIRC	kidney renal clear cell carcinoma
LIHC	liver hepatocellular carcinoma
LUAD	lung adenocarcinoma
LUSC	lung squamous cell carcinoma
DLBC	lymphoid neoplasm diffuse large b-cell lymphoma
MESO	mesothelioma
mIHC	multiplex immunofluorescence staining
MAF	mutation annotation format
NMF	negative matrix factorization
OV	ovarian serous cystadenocarcinoma
PCPG	pheochromocytoma and Paraganglioma
PRAD	prostate adenocarcinoma
READ	rectum adenocarcinoma
SARC	sarcoma
SMGs	significantly mutated genes
SKCM	skin cutaneous melanoma
SCNAs	somatic copy number alterations
STAD	stomach adenocarcinoma
TGCT	testicular germ cell tumors
TCGA	the Cancer Genome Atlas
THCA	thyroid carcinoma
TME	tumor microenvironment
TMB	tumor mutational burden
TNB	tumor neoantigen burden
UCS	uterine carcinosarcoma
UCEC	uterine corpus endometrial carcinoma
UVM	uveal melanoma
VEP	variant effect predictor
WES	whole exome sequencing
