# Supplementary figures and images for: Neoadjuvant PD-1 Blockade Combined With Chemotherapy Followed by Concurrent Immunoradiotherapy in Locally Advanced Anal Canal Squamous Cell Carcinoma Patients: Antitumor Efficacy, Safety and Biomarker Analysis
Source: Front Immunol. 2022 Jan 14;12:798451. doi: 10.3389/fimmu.2021.798451 (PMC8794813; doi:10.3389/fimmu.2021.798451)

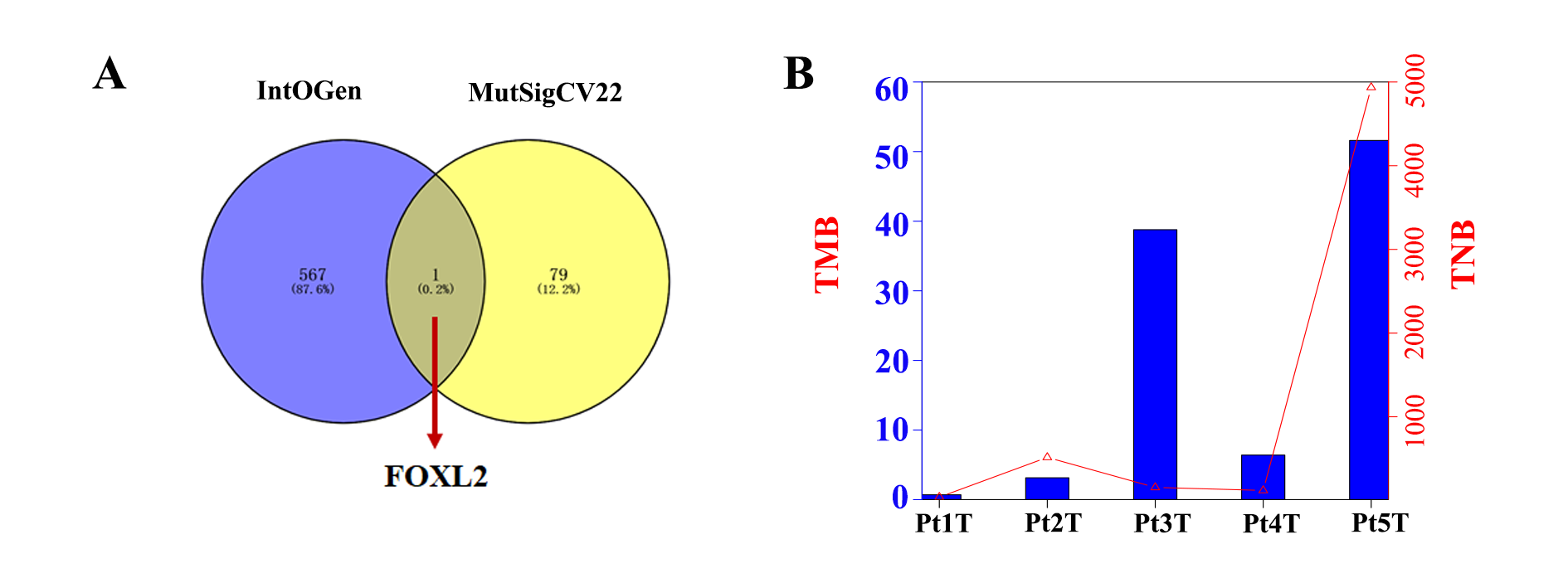

Supplement: Supplementary Figure 1 — Treatment responses after neoadjuvant treatment for patients 2, 3, 4 and 5. Colonoscopy and T2WI MRI images of the primary tumor before and after neoadjuvant treatment. [file Image_1.tif]

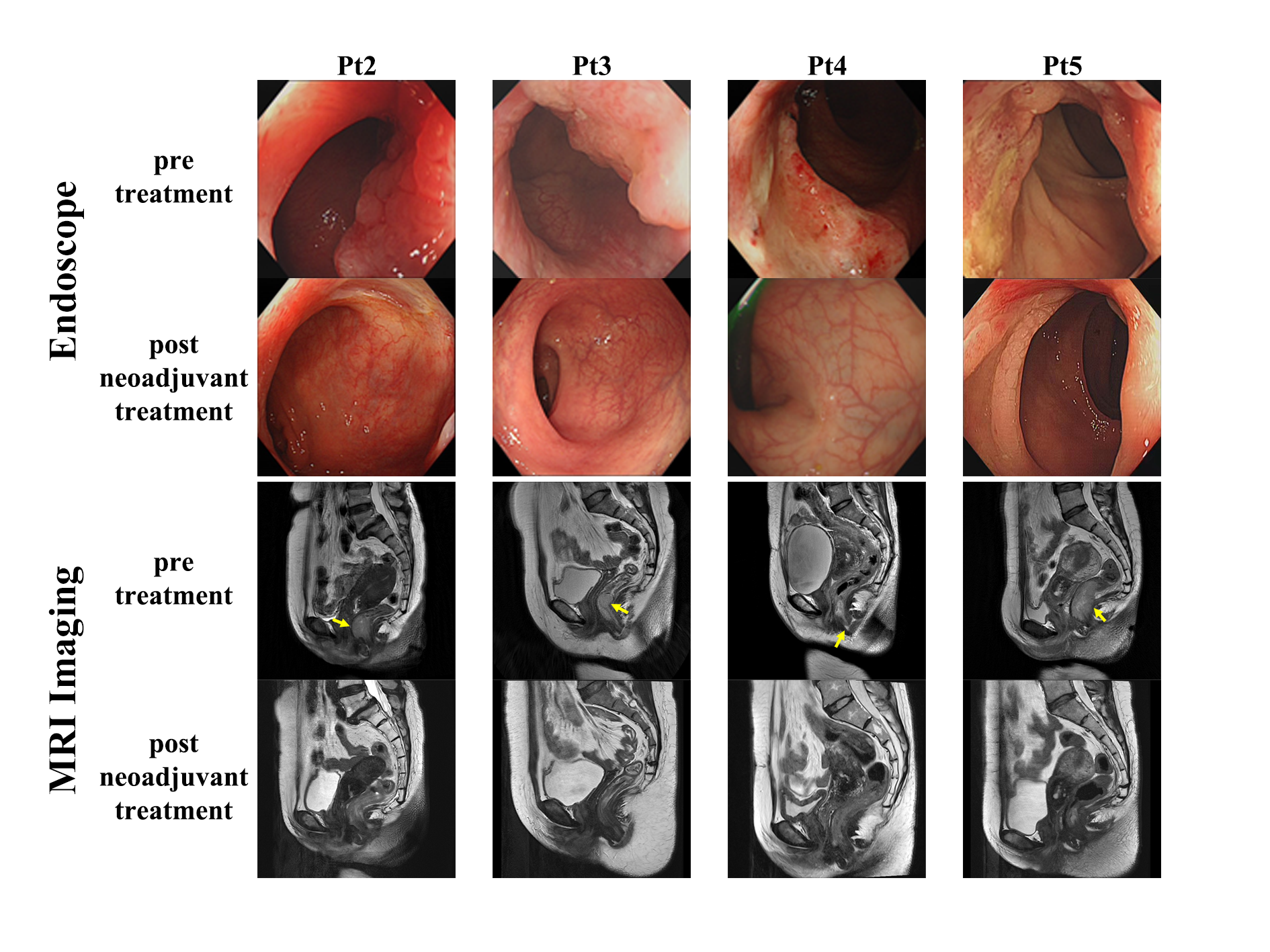

Supplement: Supplementary Figure 2 — SDGs, TMB, TNB of the five ACSCC patients. (A) SDGs identified using the IntOGen-mutations pipeline and MutSigCV and the common mutation gene FOXL2. (B) TMB, TNB of the 5 ASCS. [file Image_2.tif]
